# Preclinical immunogenicity testing using anti-drug antibody analysis of GX-G3, Fc-fused recombinant human granulocyte colony-stimulating factor, in rat and monkey models

**DOI:** 10.1038/s41598-021-91360-7

**Published:** 2021-06-07

**Authors:** Yun Jung Kim, Eun Mi Koh, Chi Hun Song, Mi Sun Byun, Yu Ri Choi, Eun-Jeong Jeon, Kyunghwa Hwang, Sang Kyum Kim, Sang In Yang, Kyung Jin Jung

**Affiliations:** 1 Genexine, Inc, Korea Bio Park, Seongnam, 13488 Republic of Korea; 2 Bioanalytical and Immunoanalytical Research Group, Department of Advanced Toxicology Research, Korea Institute of Toxicology, 141 Gajeong-ro, Yuseong-gu, Daejeon, 34114 Republic of Korea; 3 College of Pharmacy, Chungnam National University, 99 Daehak-ro, Yuseong-gu, Daejeon, 34131 Republic of Korea; 4 Jeonbuk Analytical Research Group, In Vivo Hazard Evaluation and Research Division, Jeonbuk Branch Institute, Korea Institute of Toxicology, Jeongeup, Jeollabuk-do 56212 Republic of Korea

**Keywords:** Antibody generation, Immunological disorders, Risk factors

## Abstract

Human granulocyte colony-stimulating factor (G-CSF, this study used Fc-fused recombinant G-CSF; GX-G3) is an important glycoprotein that stimulates the proliferation of granulocytes and white blood cells. Thus, G-CSF treatment has been considered as a crucial regimen to accelerate recovery from chemotherapy-induced neutropenia in cancer patients suffering from non-myeloid malignancy or acute myeloid leukemia. Despite the therapeutic advantages of G-CSF treatment, an assessment of its immunogenicity must be performed to determine whether the production of anti-G-CSF antibodies causes immune-related disorders. We optimized and validated analytical tools by adopting validation parameters for immunogenicity assessment. Using these validated tools, we analyzed serum samples from rats and monkeys injected subcutaneously with GX-G3 (1, 3 or 10 mg/kg once a week for 4 weeks followed by a 4-week recovery period) to determine immunogenicity response and toxicokinetic parameters with serum concentration of GX-G3. Several rats and monkeys were determined to be positive for anti-GX-G3 antibodies. Moreover, the immunogenicity response of GX-G3 was lower in monkeys than in rats, which was relevant to show less inhibition of toxicokinetic profiles in monkeys, at least 1 mg/kg administrated group, compared to rats. These results suggested the establishment and validation for analyzing anti-GX-G3 antibodies and measurement of serum levels of GX-G3 and anti-GX-G3 antibodies, which was related with toxicokinetic profiles. Taken together, this study provides immunogenicity assessment which is closely implicated with toxicokinetic study of GX-G3 in 4-week repeated administrated toxicological studies.

## Introduction

GX-G3 was developed as a fusion protein of granulocyte-colony stimulating factor (G-CSF) with the hybrid-Fc (hyFc) platform proprietary to Genexine, Inc.; GX-G3 is expected to be a candidate best-in-class drug.

Many candidate recombinant protein therapeutics have been investigated, and recombinant cytokines were endorsed for the prevention of bacterial infection in immune-compromised hosts due to their modulation of immunity^1^. Among these cytokines, colony-stimulatory factors induce bone marrow to produce leukocytes that fight infectious diseases. Particularly, G-CSF was considered worthy of investigation as a biotherapeutic protein because G-CSF is an endogenous hematopoietic growth factor that controls the production, differentiation and function of neutrophil precursor granulocytes from bone marrow and increases the survival and activity of mature neutrophils^2–4^. A recombinant form of human G-CSF, specifically the recombinant methionyl human G-CSF NEUPOGEN® (filgrastim), was initially approved with an indicated use to decrease the incidence of infection and reduce severe neutropenia. Subsequently, filgrastim was also approved for use in patients suffering from acute myeloid leukemia (AML), severe congenital neutropenia, AIDS-associated neutropenia, or nonmyeloid malignancies receiving myelosuppressive anti-cancer drugs^5–7^. Since then, a PEGylated form of filgrastim, pegfilgrastim, was developed to prolong the plasma half-life and improve the dosing regimen; this drug is currently marketed and approved for the prevention of chemotherapy-induced neutropenia^8,9^.

Additional modifications of G-CSF therapeutics continue to be investigated to produce better drugs. The most clinically advanced non-PEGylated form of G-CSF is benefilgrastim, which is a fusion protein of human Fc with G-CSF produced in mammalian cells^10^. Benefits of benefilgrastim have been reported in a clinical phase II study during multiple chemotherapy cycles (Glaspy et al., 2014). Fc-containing fusion protein drugs predominantly target receptor-ligand interaction by acting as either antagonists to block receptor binding or agonists to directly stimulate receptor function, thereby reducing or increasing immune activity, respectively^11^. For bioconjugation of Fc, human IgG1 Fc has been widely used, although it has disadvantages such as antibody-dependent cell cytotoxicity (ADCC) and complementary-dependent cytotoxicity (CDC)^12^. For the generation of GX-G3, a proprietary Fc-fusion technology of Genexine Inc. termed “hyFc” that was constructed via nonimmunogenic, noncytolytic and flexible Fc conjugate consisting of IgD and IgG4 was applied. The aim of GX-G3 development was to extend the drug half-life and eliminate unwanted ADCC or CDC^13,14^.

Although biotherapeutics have many benefits compared to small molecule drugs with respect to disease target selectivity, unwanted immunogenicity can still be a hurdle to the development of biotherapeutics and the establishment of the appropriate dosages^15^. Because the production of anti-drug antibodies (ADAs) against biotherapeutics can have detrimental effects on drug safety, efficacy, and pharmacokinetics, immunogenicity an important factor in the toxicity profile of biotherapeutic proteins^16^. Some evidence indicates that protein aggregation, the route of administration, patient-related factors, or chemistry, manufacturing and control (CMC) can impact immunogenicity; however, the precise immunological and biochemical mechanisms responsible for the immunogenicity of biotherapeutics are poorly understood^17^. Moreover, the results from various studies are difficult to compare because of a lack of standardization of the ADA assays, often resulting in contradictory findings. Despite this limitation, the monitoring and interpretation of drug immunogenicity resulting from ADA production are very important throughout the in-study phase. Although immunogenicity to biologics is often not directly associated with adverse events in animal models, these studies have limited predictive power for the immunogenicity of the biologic in humans. ADA assays can reflect certain aspects of drug bioavailability, neutralizing effects, and endogenous cross-reactivity, reflecting significant safety concerns^18^. ADAs can also cause adverse events, including administration reactions such as systemic infusion reactions, localized injection reactions or acute hypersensitivity reactions^19^.

The assessment of the immunogenicity of recombinant therapeutic proteins (RPTs) in animal studies could provide evidence to predict the induction of clinically adverse events in RPT-treated patients and to determine the relative immunogenicity of RTPs in animals that optimizes an immunological response. Therefore, tools should be developed to detect ADAs and should be pre-validated by optimizing validation parameters such as negative cut-off value, precision, sensitivity, specificity confirmation, and interference by the matrix or the study drug in the preclinical and clinical phases, respectively^20^.

In this study, we performed experiments to measure the serum concentration of GX-G3 and titer quantity of GX-G3-specific antibodies in rats and monkeys induced by repeated administration of GX-G3. Here, we present the validated tool for the detection of anti-GX-G3 antibodies and report a potential immunogenicity which affects to GX-G3 toxicokinetic data in nonclinical rat and monkey studies subcutaneously administrated with GX-G3 for 4 weeks followed by a 4-week recovery period.

## Results

### Determination of NCO values for anti-GX-G3 antibody measurement

**Figure 1:**
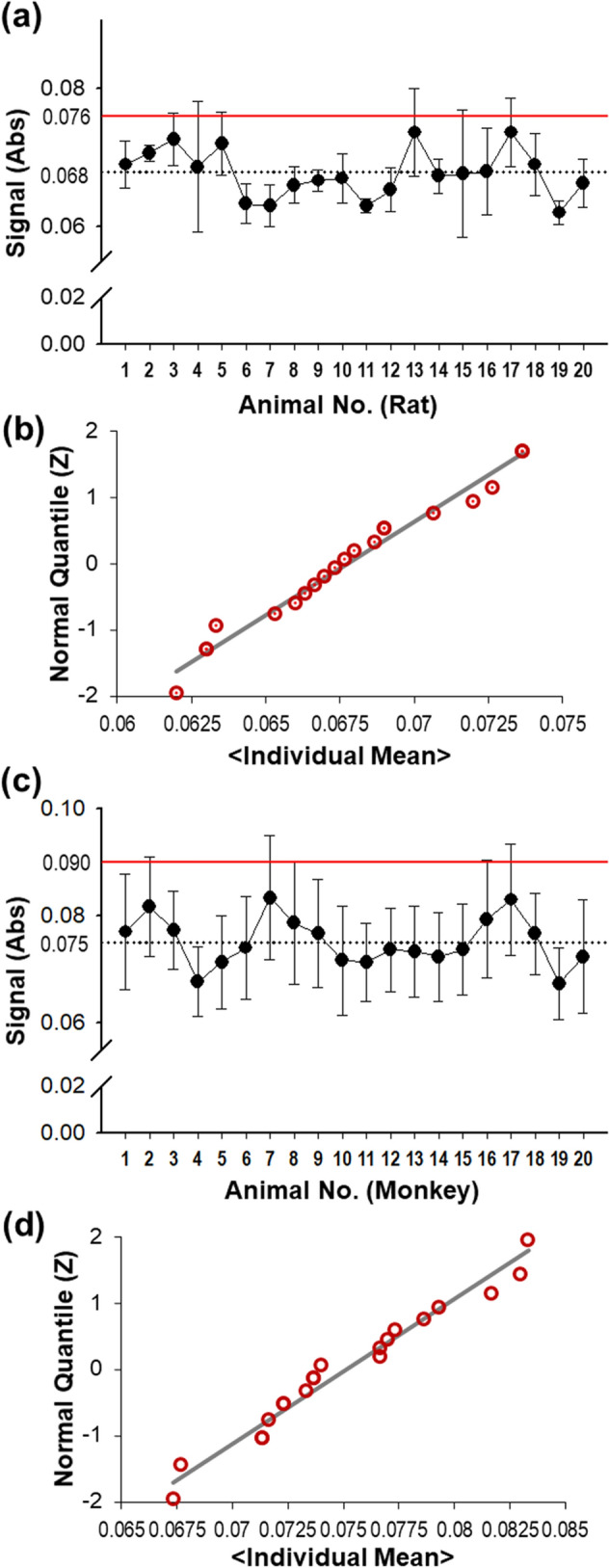
Negative cut-off (NCO) values were determined using 20 blank serum samples from rats and monkeys. For blank rat sera, (**a**) determination of the NCO value, (**b**) normal Q-Q plot of individual data. For blank monkey sera, (**c**) determination of the NCO value, (**d**) normal Q-Q plot of individual data.

Negative blank absorbance values of 20 individual sera in rats were measured using an anti-GX-G3 bridging ELISA method in three independent assays to collect baseline absorbance values (Fig. 1A). Among the measured values, box plot analysis was used to identify outliers, but no outliers were identified (data not shown). The measured values were calculated to establish 95% confidence of 5% false positives as previously recommended by Mire-Sluis et al.^20^. The mean absorbance value of naïve blank sera was 0.068 OD and the mean NCO value was 0.076 OD based on the assay validation experiments in rats. The W value results of the Shapiro–Wilk test for the 20 rat serum samples was 0.96, with acceptable skewness (0.17) and kurtosis (− 0.72); these results verified the normality of the distribution of absorbance values in the quantile–quantile plot (Fig. 1B). In monkeys, negative blank absorbance values of 20 individual sera were measured using the anti-GX-G3 bridging ELISA method, and the OD values were statistically analyzed to identify the mean OD of negative blank sera (0.075) and the NCO value (0.090) (Fig. 1C). Subsequently, box plot analysis was performed, and the test for outliers was performed (no outliers were identified, data not shown). The W value results of the Shapiro–Wilk test for the samples from monkeys were 0.96, with acceptable skewness (0.23) and kurtosis (− 0.52); these results verified the normality of the distribution of absorbance values in the quantile–quantile plot (Fig. 1D).

### Assay precision and sensitivity for anti-GX-G3 antibody measurement

**Figure 2:**
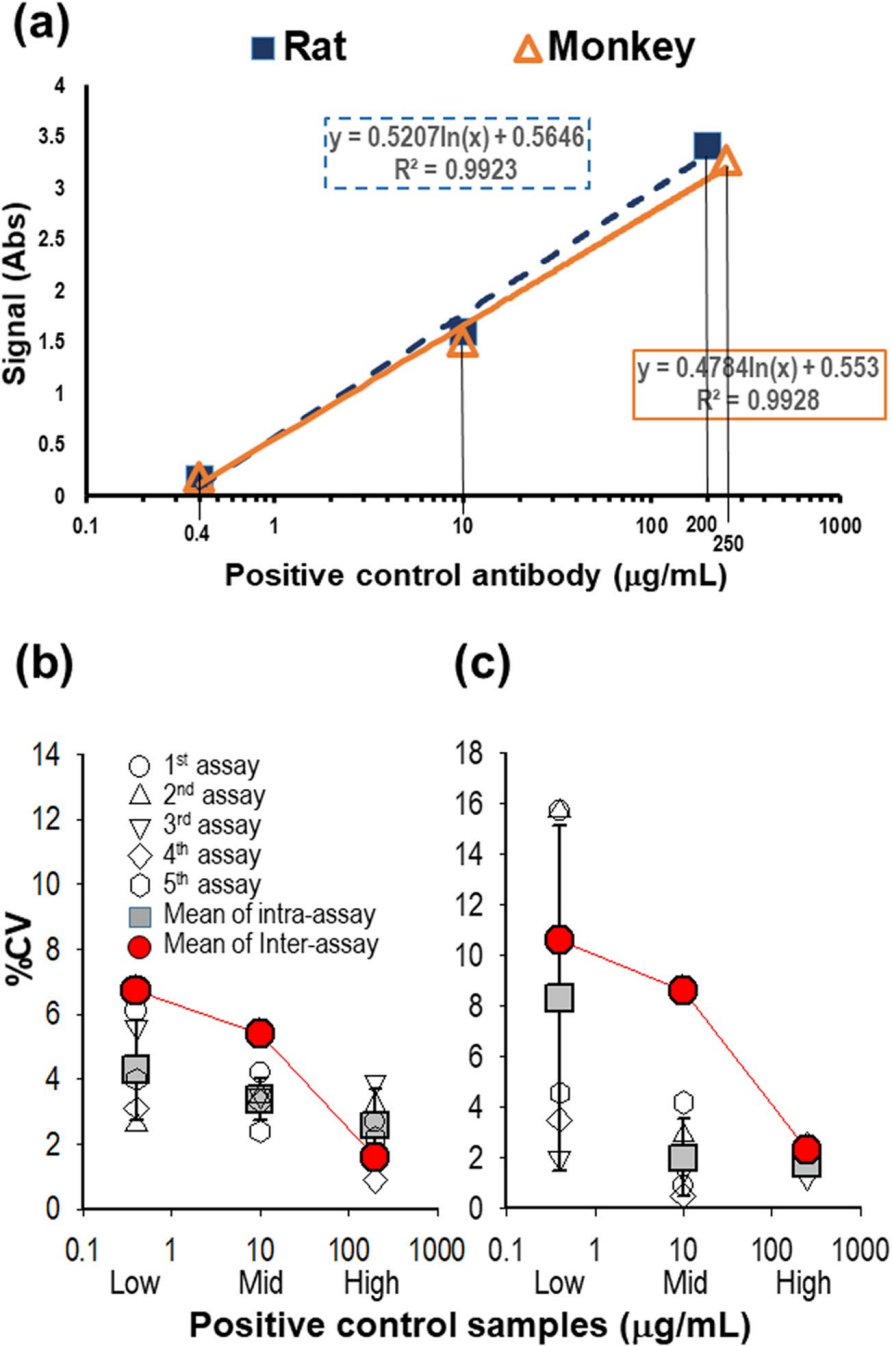
Evaluation of assay precision. Intra- and inter-assay variability were estimated using the %CV in 5 independent assays. (**a**) Representative high, middle, and low concentrations of the positive control antibody were selected in the linear range of a sigmoid curve for each set of serum samples. (**b**) High (200 μg/mL), middle (10 μg/mL), and low (0.4 μg/mL) concentrations of positive control samples (PC) in rat sera were measured for assay precision. (**c**) High (250 μg/mL), middle (10 μg/mL), and low (0.4 μg/mL) concentrations of PC in monkey sera were measured for assay precision.

Pooled rat sera were spiked with 0.4, 10, or 200 μg/mL positive control antibody, and pooled monkey sera were spiked with 0.4, 10, and 250 μg/mL positive control antibody. Each concentration of positive control samples was selected to represent low, middle, and high concentrations of ADA-positive samples from the linear region of each logistic regression curve (Fig. 2A). These positive control samples were used for evaluating assay precision. Five independent assays were performed using positive control samples, and the precision, expressed as %CV, was calculated within and between assays. For rat sera, intra-assay precision ranged from 0.9% to 6.1%, and inter-assay precision ranged from 1.6% to 6.7% between assays (Fig. 2B). For monkey sera, intra-assay precision ranged from 0.4% to 15.7%, and inter-assay precision ranged from 2.3% to 10.6% (Fig. 2C). The absorbance values and signal-to-NCO ratios, as an alternative to signal-to-noise ratios, are presented in Table 1. Also, the sample stability was evaluated under five cycles of repeated-freeze/thaw and 12-week storage condition at -80 °C. All stability samples were qualified by the result that there were no significant difference compared to the result in precision (Table 1).

**Table 1:** Repeatability (intra-assay precision) and reproducibility (inter-assay precision).

			High PC	Middle PC	Low PC	Negative cut-off
Intra-assay	Rat	Mean (%CV)	3.337 (2.6%)	1.512 (3.4%)	0.150 (4.3%)	0.067 (6.3%)
	Monkey	Mean (%CV)	3.204 (1.8%)	1.367 (2.0%)	0.178 (8.3%)	0.083 (9.7%)
Inter-assay	Rat	Mean (%CV)	3.414 (1.6%)	1.618 (5.4%)	0.169 (6.7%)	0.068 (4.6%)
		Ratio (%CV)^a^	49.9 (5.1%)	23.7 (5.8%)	2.5 (6.2%)	−
	Monkey	Mean (%CV)	3.255 (2.3%)	1.469 (8.6%)	0.183 (10.6%)	0.086 (4.0%)
		Ratio (%CV)	37.9 (3.9%)	17.1 (9.4%)	2.1 (11.7%)	−
Stability (freeze/thaw)	Rat	Mean	3.306	NA^c^	0.179	0.072
		Ratio (%difference)^b^	45.9 (8.0%)	NA	2.5 (0%)	−
	Monkey	Mean	3.287	NA	0.185	0.088
		Ratio (%difference)	37.4 (1.3%)	NA	2.1 (0%)	−
Stability (12-week)	Rat	Mean	3.431	NA	0.208	0.069
		Ratio (%difference)	49.7 (0.4%)	NA	3.0 (20%)	−
	Monkey	Mean	3.360	NA	0.206	0.084
		Ratio (%difference)	40 (5.5%)	NA	2.5 (19%)	−

^a^Ratio of absorbance of the PC to absorbance of the negative cut-off.

^b^Absolute value of %difference from precision.

^c^Not applied.

**Figure 3:**
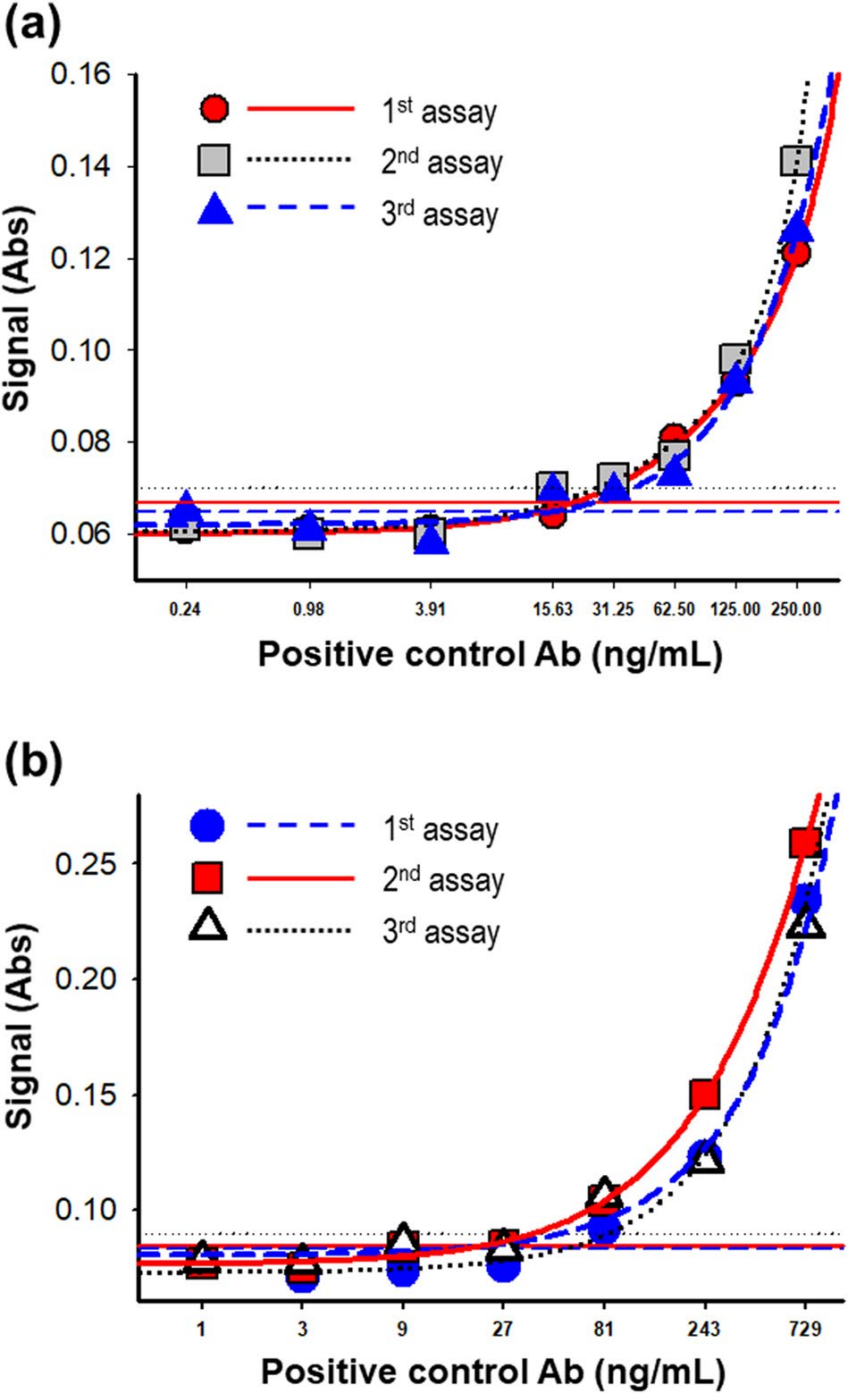
Estimation of assay sensitivity. (**a**) Assay sensitivity was measured in rat sera. PC were spiked into pooled rat serum samples at concentrations ranging from 0.24 to 250 ng/mL. (**b**) For monkey serum, PC were prepared at concentrations ranging from 1 to 729 ng/mL. The lowest concentration at which the absorbance value was equal to the normalized NCO value in each trial is shown for each sensitivity assay.

The assay sensitivity was measured using multiple dilutions of the positive control antibody in pooled rat or monkey serum samples using 4-parameter logistic curve-fitting. The mean assay sensitivity for rat serum samples was 22.8 ng/mL (the average of 13.8, 22.5, and 32.1 ng/mL from 3 independent assays) (Fig. 3A). The mean assay sensitivity for monkey serum samples was 40.8 ng/mL (the average of 20.0, 50.1, 52.3 ng/mL from 3 independent assays) (Fig. 3B).

### Specificity and drug interference of GX-G3

**Figure 4:**
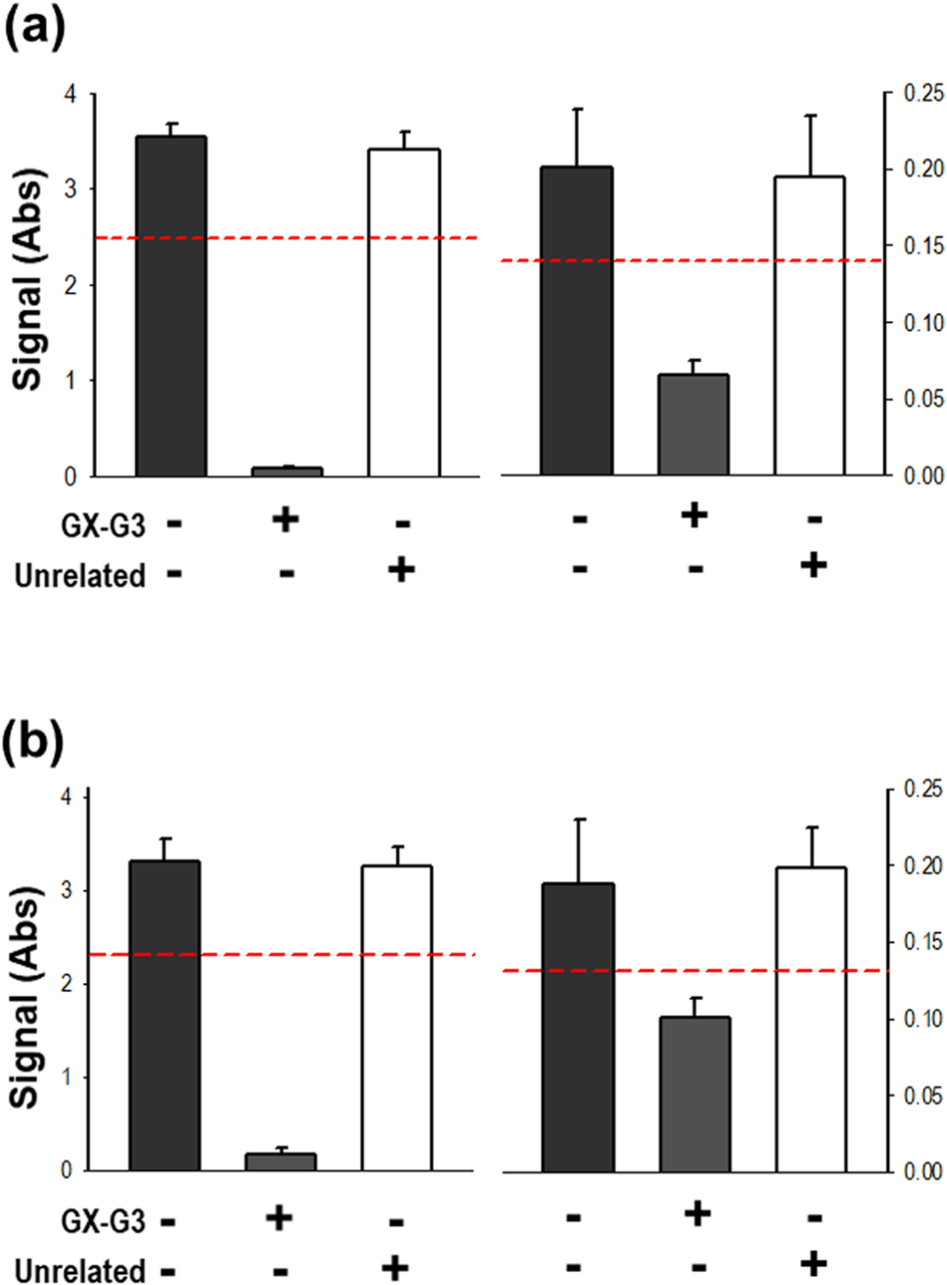
Specificity confirmation assay. PC were characterized by testing immune-competition for specificity confirmation. High- and low-concentration PC were pre-incubated for 1 h with the drug GX-G3 or a comparator molecule considered to be unrelated to G-CSF binding, such as rh-GH. (**a**) High- and low-concentration PC were spiked into pooled rat serum samples at concentrations of 200 μg/mL and 0.4 μg/mL, respectively. (**b**) High- and low-concentration PC were spiked into pooled monkey serum samples at concentrations of 250 μg/mL and 0.4 μg/mL, respectively. High- and low-concentration PC were pre-incubated with 150 μg/mL GX-G3 for rat serum or 200 μg/mL GX-G3 for monkey serum. rh-GH was used as an unrelated antigen and was spiked into the samples at the same concentrations as GX-G3. High-concentration PC, high-concentration positive control samples; Low-concentration PC, low-concentration positive control samples; Unrelated, unrelated antigen; rh-GH, recombinant human growth hormone.

For confirmation of assay specificity, high- and low-concentration ADA–containing PC in rat and monkey sera were spiked with GX-G3 antigen or the unrelated comparator human growth hormone (hGH)-hyFc or hyFc, followed by incubation at 23 °C for 1 h. Afterwards, the levels of ADAs were measured, and the %difference was analyzed by applying the 30% cut-off value for the difference in specificity compared to the non-antigen-spiked PC. In rat sera, PC treated with GX-G3 displayed 92% and 67.9% differences in absorbance relative to the high- and low-concentration PC, respectively; thus, these values were greater than the specificity cut-off value. However, PC treated with the unrelated molecule hGH-hyFc or hyFc showed no significant difference in absorbance compared with the untreated PC; thus, these values were less than the specificity cut-off value (Fig. 4A). Additionally, in monkey sera, PC treated with GX-G3 displayed greater differences relative to the high- and low-concentration PC than the specificity cut-off value; however, PC treated with either unrelated molecule showed no difference in absorbance compared with the untreated PC (Fig. 4B). These data indicated the specificity of GX-G3 against ADAs, indicating that this assay could detect and confirm the presence of specific anti-GX-G3 antibodies.

**Figure 5:**
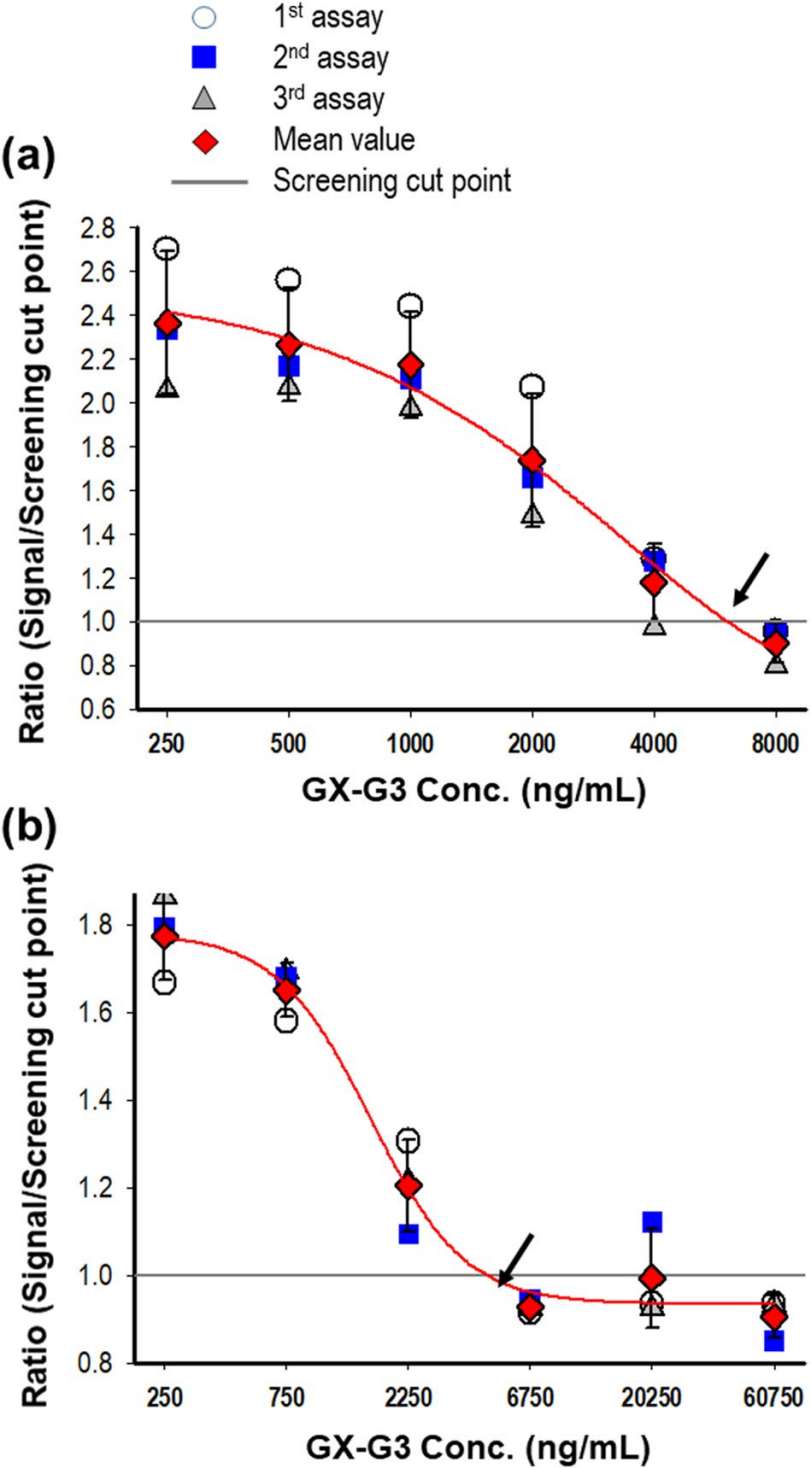
Estimation of drug interference. Low-concentration PC were pre-incubated with serial dilutions of the drug GX-G3 for 1 h. (**a**) Low-concentration PC in pooled rat sera were pre-incubated with the drug GX-G3 at a concentration ranging from 250 to 8000 ng/mL. (**b**) Low-concentration PC in pooled monkey sera were pre-incubated with the drug GX-G3 at a concentration ranging from 250 to 60750 ng/mL. The highest concentration of drug at which the absorbance value was equal to the normalized NCO value is shown in the graphs.

To assess drug interference in the ADA detection assay, low-concentration PC in rat and monkey sera were spiked with GX-G3 antigen at serial dilutions of 250 − 8000 ng/mL. The results of curve-fitting (GX-G3 concentration vs. absorbance) using a four-parameter regression model are shown. According to the curve-fitting with proper correlation coefficient (R^2^ > 0.99), drug interference was identified as the concentration of GX-G3 at which the OD value was equal to the normalized NCO value. The observed drug interference limits using the low-concentration PC, represent samples with low levels of ADAs, were 6610 ng/mL for rat serum samples and 4584 ng/mL for monkey serum samples (Fig. 5A,B).

### Immunogenicity assessment for GX-G3-administered rats and monkeys

To evaluate the immunogenicity of GX-G3 in a 4-week repeated-dose toxicity study, rats and monkeys were treated once a week via subcutaneous injection of 0 (vehicle control) 1, 3, or 10 mg/kg GX-G3. Serum samples were tested via the aforementioned, validated bridging ELISA method.

**Figure 6:**
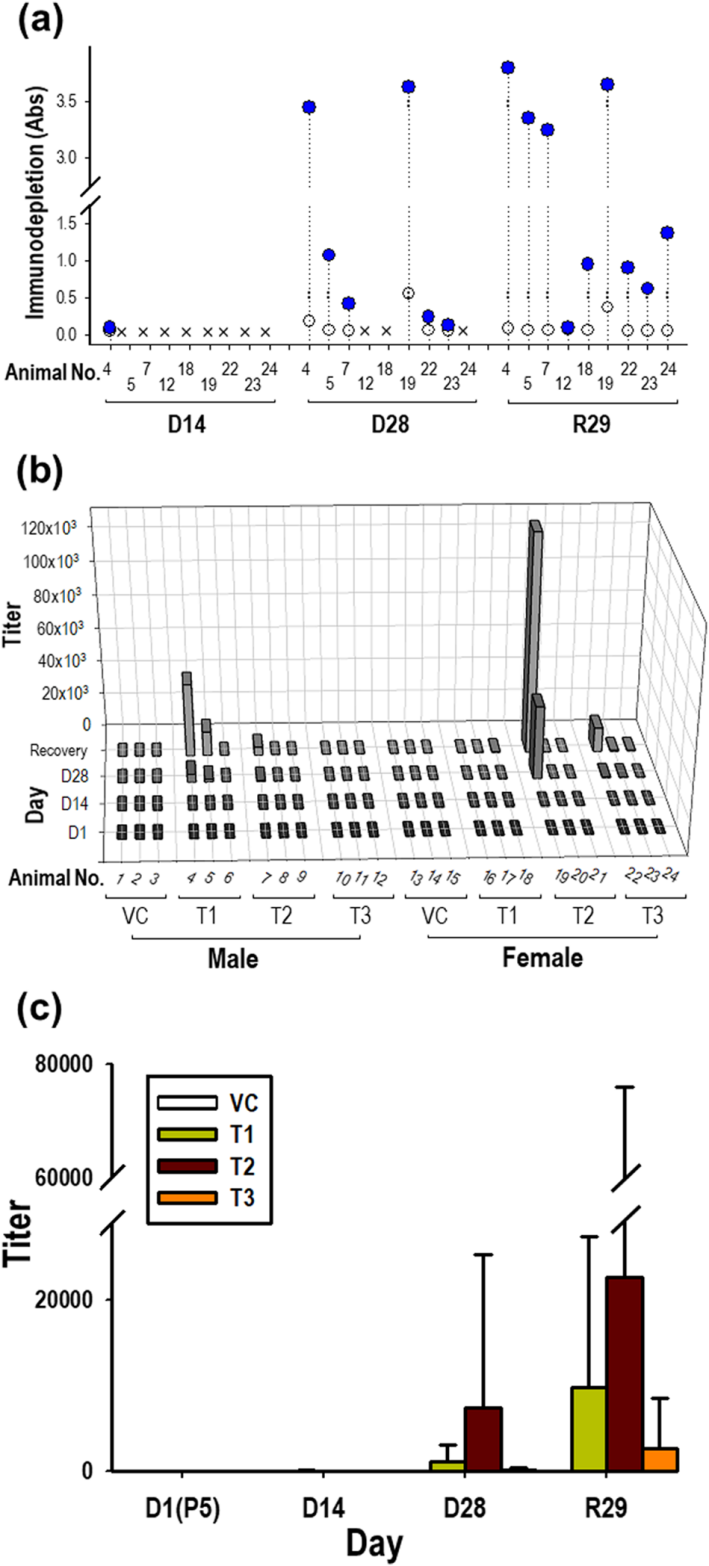
Measurement of ADAs, representing anti-GX-G3 antibodies, in rats subjected to 4-week repeated subcutaneous injection with GX-G3 followed by a 4-week recovery period. Rats were separated into 4 injection subgroups: VC (vehicle control), T1 (1 mg/kg GX-G3), T2 (3 mg/kg GX-G3), and T3 (10 mg/kg GX-G3). ADA levels in rat sera were measured using the method validated in the present study. (**a**) Immuno-competition assays were performed to discriminate between true and false positive samples. True positivity for GX-G3 specificity was identified based on a greater than 30% difference in absorbance between the non-treated and GX-G3-treated samples. (**b**) Rat serum samples determined to be true positives were prepared as serial three-fold dilutions beginning from 20-fold. The antibody titer of each sample was expressed as the highest fold-dilution displaying a positive response (absorbance value > normalized NCO value). (**c**) The mean antibody titer of each group was arithmetically calculated. VC, vehicle control group; P5, pre-dosing day 5; D14 and D28, dosing days 14 and 28, respectively; R29, recovery day 29.

In rats, 22 out of the 96 collected samples were initially screened to determine their positivity for GX-G3 antibodies based on the detection of a greater absorbance value than the NCO value. After screening, a confirmation assay was performed to discriminate whether the samples identified as potentially positive for GX-G3 antibodies based on the screening assay were true or false positives. True positivity was confirmed in 16 samples, according to a greater than 30% difference in absorbance (specificity cut-off value) compared to the GX-G3-spiked samples. Antibody positivity was observed beginning on dosing day 14, and 1/24, 6/24, and 9/24 animals were determined as true positives on dosing day 14, dosing day 28, and recovery day 29, respectively (Fig. 6A). To establish the antibody titer, individual samples determined as true positives were serially diluted by 20- to 393660-fold (Fig. 6B). On dosing day 28, the mean antibody titers were 5130, 7830, and 570 in the 1, 3, and 10 mg/kg GX-G3-treated groups, respectively. Over the recovery period, there was a tendency toward an increase in antibody titers on recovery day 29 compared to those on dosing day 28 (Fig. 6C). There was no significant difference in antibody titers between GX-G3 doses or sampling days because large variation in the data existed.

**Figure 7:**
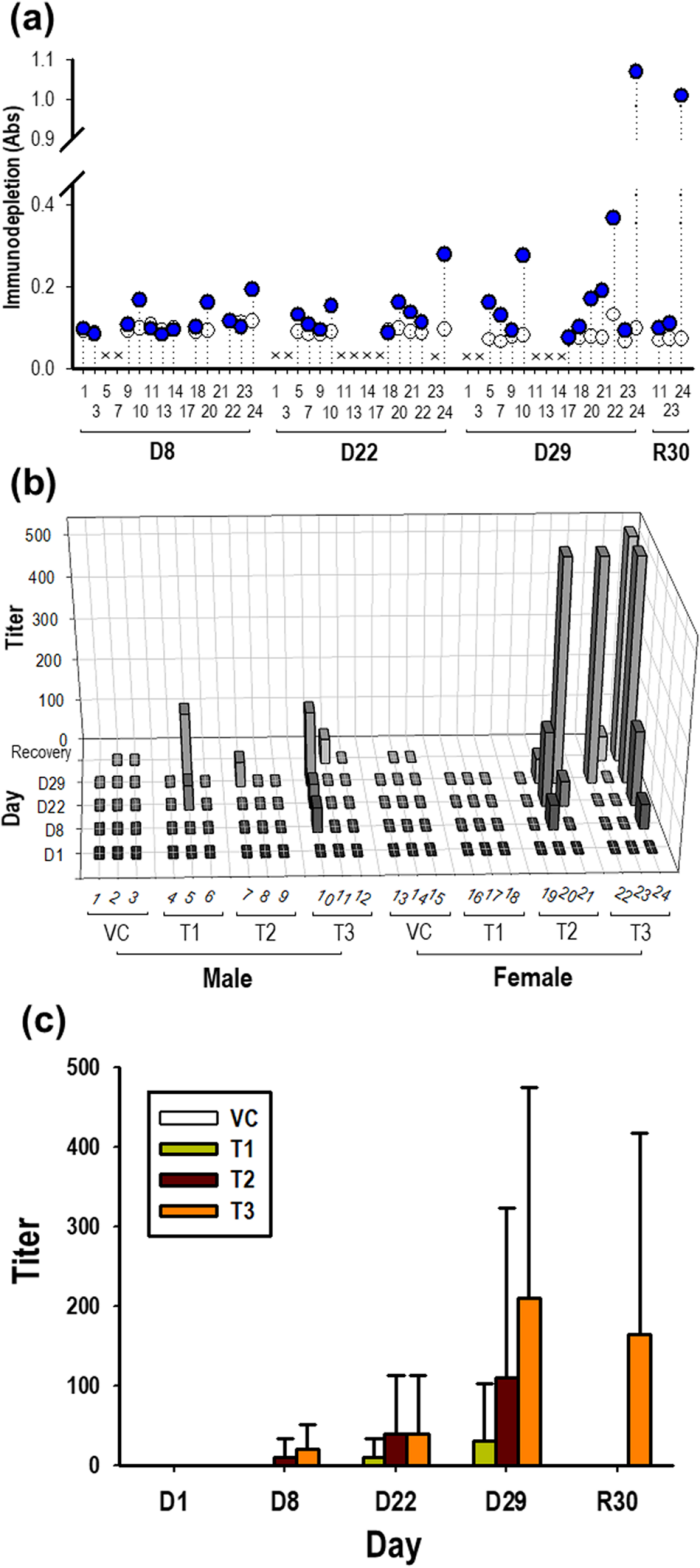
Measurement of ADAs, representing anti-GX-G3 antibodies, in monkeys subjected to 4-week repeated subcutaneous injection with GX-G3 followed by a 4-week recovery period. Monkeys were separated into 4 injection subgroups: VC (vehicle control), T1 (1 mg/kg GX-G3), T2 (3 mg/kg GX-G3), and T3 (10 mg/kg GX-G3). ADA levels in monkey sera were measured using the method validated in the present study. (**a**) Immuno-competition assays were performed to discriminate between true and false positive samples. True positivity for GX-G3 specificity was identified based on a greater than 30% difference 30% in absorbance between the non-treated and GX-G3-treated samples. (**b**) Monkey serum samples determined to be true positives were prepared as serial three-fold dilutions beginning from 20-fold. The antibody titer of each sample was expressed as the highest fold-dilution displaying a positive response (absorbance value > normalized NCO value). (**c**) The mean antibody titer of each group was arithmetically calculated. VC, vehicle control group; D1, D8, D22 and D29, dosing days 1, 8, 22 and 29, respectively; R30, recovery day 30.

In monkeys, 35 out of the 104 collected samples were first screened to determine their positivity for GX-G3 antibodies based on the detection of a greater absorbance value than the NCO value. After screening, a confirmation assay was performed to discriminate whether the samples identified as potentially positive based on screening assay were true or false positives. True positivity was confirmed for 18 samples, according to a greater than 30% difference in absorbance (specificity cut-off value) compared to the GX-G3-spiked samples. Antibody positivity was observed beginning on day 8, and 3/24, 5/24, 7/24 and 3/8 animals were determined as true positives on dosing day 8, dosing day 22, dosing day 29 and recovery day 30, respectively (Fig. 7A). To determine antibody titers, individual samples determined to be true positives were serially diluted by 20- to 393,660-fold (Fig. 7B). On day 29, the mean antibody titers were 30, 110, and 210 in the 1, 3, and 10 mg/kg GX-G3-treated groups, respectively. There was no significant difference in antibody titer between GX-G3 doses or sampling days because large variation in the data existed. After the recovery period, several samples showed increased antibody titers in the highest dosing group, but the maximum titer in any individual monkey serum sample was 540 following the recovery period (Fig. 7C). As shown in Figs. 6C and 7C, the mean antibody titers of monkey serum samples over the recovery period for monkeys were much lower than those of rat serum samples.

### Effect of immunogenicity on preclinical toxicokinetic profiles of GX-G3

**Figure 8:**
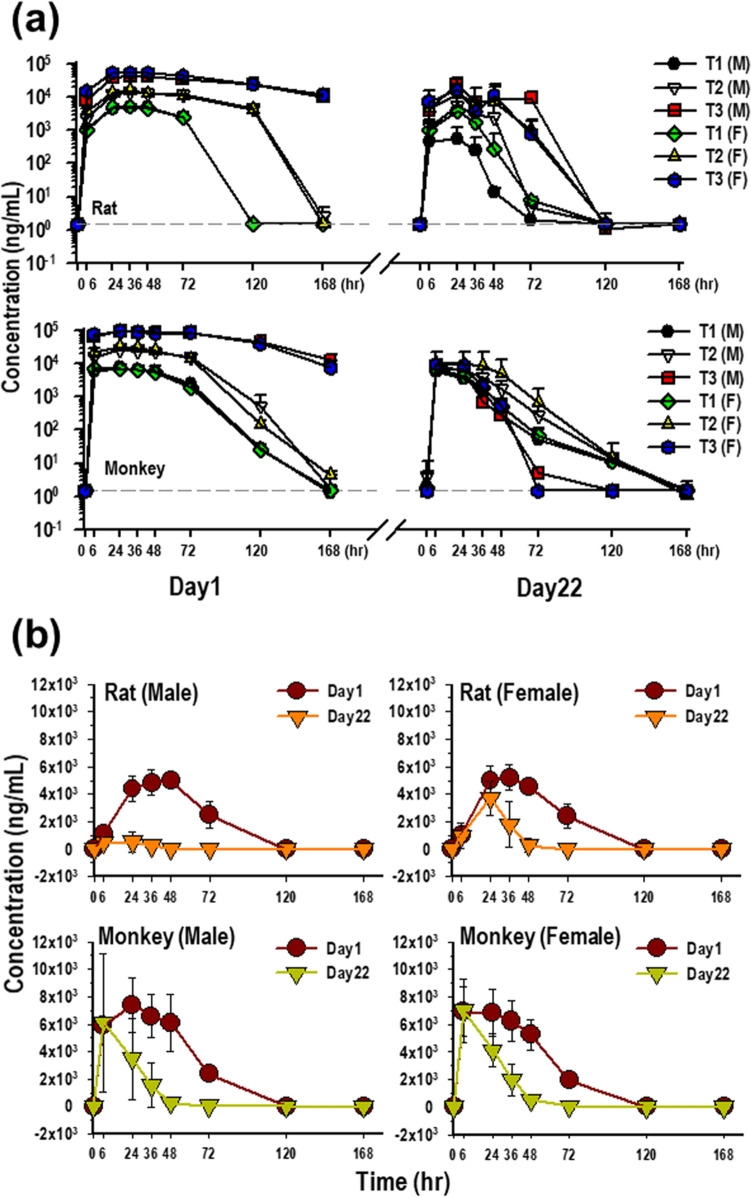
Evaluation of the impact of immunogenicity on GX-G3 toxicokinetic analysis, in rats and monkeys subjected to 4-week repeated subcutaneous injection with GX-G3 followed by a 4-week recovery period. Each species was separated into 4 injection subgroups: VC (vehicle control), T1 (1 mg/kg GX-G3), T2 (3 mg/kg GX-G3), and T3 (10 mg/kg GX-G3). (**a**) Measurement of GX-G3 concentration–time profiles after subcutaneous administration of GX-G3 on Day 1 (Week 0) and Day 22 (Week 3) in rats and monkeys. (**b**) Representative concentration–time profile of T1 group on Day 1 and Day 22 in rats and monkeys.

The concentration of GX-G3 obtained for each serum sample was obtained with bioanalytical method showing lowest quantifiable limit 1.5 ng/mL. The toxicokinetic profiles of GX-G3 after repeated administration of subcutaneous doses (1, 3, and 10 mg/kg) on Day 1 and Day 22 in rats and monkeys are shown in Fig. 8A, and TK parameters are summarized in Table 2. After the first administration on Day 1, GX-G3 reached maximum serum concentration C_max_ and then was eliminated from serum in rats and monkeys, which showed the increase with an approximately dose proportional manner in 1–10 mg/kg subcutaneous dose range in rats and monkeys. T_1/2_ ranged from 31.16 to 61.14 h in rats and 8.29 to 34.76 h in monkeys. CL values ranged from 1.76 to 3.15 mL/h/kg in rats and 0.99 to 2.63 mL/h/kg in monkeys (Table 2). In toxicokinetic analysis on Day 22, all parameters, except for CL, were much lower than that seen at Day 1 without relevance of dose proportional manner. Also, the trough concentration was not significantly increased at Day 22 compared with Day 1 at all doses. The difference of kinetics between Day 1 and Day 22 for single dose and repeated dose, respectively, was more easily shown in Fig. 8B which shows only 1 mg/kg administrated group. Thus, these results indicated that the formation and increase of anti-drug antibody adversely affected to the investigation of drug exposure and toxicokinetic analysis.

**Table 2:** Toxicokinetic parameters in Sprague Dawley rats and Cynomolgus monkeys administrated with GX-G3.

Day of analysis	Parameters	Sex	Rat			Monkey		
			Dose (mg/kg)			Dose (mg/kg)		
			1	3	10	1	3	10
Day 1	T_1/2_ (h)	Male	35.00	46.81	61.14	9.30	9.16	34.76
		Female	31.16	44.77	49.46	9.12	8.29	23.97
	C_max_ (μg/mL)	Male	4.99	14.79	43.54	7.42	27.02	96.51
		Female	5.21	15.85	54.91	7.12	32.82	100.50
	AUC_inf_ (h*μg/mL)	Male	390.96	1286.34	5737.49	418.42	1707.83	11,071.47
		Female	372.55	1442.66	6540.30	394.52	1850.98	10,074.07
	CL (mL/h/kg)	Male	3.15	2.33	2.13	2.45	1.82	0.99
		Female	3.13	2.39	1.76	2.63	1.66	1.03
Day22	T_1/2_ (h)	Male	5.42	3.64	5.17	12.74	8.39	4.83
		Female	4.60	12.01	13.25	14.31	7.73	5.95
	C_max_ (μg/mL)	Male	0.55	5.62	25.96	6.10	7.29	9.04
		Female	3.74	11.97	16.84	7.01	11.16	9.42
	AUC_inf_ (h * μg/mL)	Male	17.12	177.62	1025.50	144.30	257.92	197.61
		Female	94.43	482.83	620.67	172.66	668.63	268.18
	CL (mL/h/kg)	Male	5.83	1.69	9.75	46.46	15.85	50.59
		Female	1.06	6.16	1.62	6.31	38.15	36.99

Toxicokinetic parameters are based on mean values from three animals per group.

T_1/2_ = terminal half-life, C_max_ = maximum serum concentration, AUC_inf_ (h * μg/mL) = area under the curve (the concentration of GX-G3 in serum vs. time) from time zero to infinity, CL = total systemic clearance after administration.

## Discussion

With the advances in molecular engineering and recombinant protein engineering technology, recombinant protein therapeutics are becoming much more common in the industrial market. However, the immunogenicity of recombinant protein therapeutics remains a major concern, and optimized tools are needed to measure ADA levels and appropriately interpret the results in drug safety assessments. Moreover, assessing immunogenicity is complex, and such assessments should account for the detection of new drug-specific responses. Thus, investigators have regarded the evaluation of immunogenicity as important for the identification of specific immunogenic epitopes and for pharmacokinetic profiling^22^.

In the present study, we developed and validated a method to detect ADAs and preliminarily evaluated ADA production to assess the immunogenicity of GX-G3, a new recombinant G-CSF-containing drug. To observe the presence of ADAs in the preclinical phase, rats and monkeys were subcutaneously injected with GX-G3 at 1, 3, or 10 mg/kg once a week for four weeks, followed by a four week recovery period. The ADA detection method of bridging ELISA was separately applied to rat and monkey sera. In both species, the validated tool satisfied the precision criteria and showed sufficient sensitivity to detect ADAs at concentrations as low as 32.1 ng/mL in rat sera and 52.3 ng/mL in monkey sera; 500–1000 ng/mL has been reported to be reasonable sensitivity in preclinical studies^20^. The specificity of the method in each species was confirmed via immune-competition assays by comparing samples treated with the drug GX-G3 with samples treated with the unrelated molecule hyFc or human growth hormone conjugated to the same Fc molecule.

In this study, we observed an interfering factor for the detection of ADAs: the drug. Drug tolerance for the low-concentration PC was established to be a free drug concentration in blood of approximately 4583.8 ng/mL for monkeys and 6610.4 ng/mL for rats. These values indicate sufficient capacity to determine whether samples are negative or positive for ADAs at certain time points of collection in the presence of the drug. This evidence showed that these detection methods are useful tools in the presence of a low residual concentration of the free drug in blood for the detection of relatively low levels of ADAs. The generated ADA also could disturb toxicokinetic analysis for the measurement of drug concentration in biological matrix. TK analysis was also performed in this study, and as a result, it was observed that there is possibility to inhibit pharmacoactive drugs (Fig. 8 and Table 2).

Importantly, the animal study showed lower levels of ADAs in monkeys than in rats. The antibody titers were different by two orders of magnitude between rats and monkeys, with a much higher titer in rats (Figs. 6 and 7). Thus, the results revealed that GX-G3, a human recombinant form of G-CSF, might be less immunogenic in monkeys than in rats due to the relatively small genetic difference between humans and monkeys compared to that between humans and rodents.

A comparison of the G-CSF protein sequence between these three species, i.e., human, monkey and rat, showed the closest homology between human and monkey G-CSF. The protein sequence homology was 94.7% between monkey and human G-CSF and was 75.9% between rat and human G-CSF. Moreover, differences in the glycosylation pattern of recombinant proteins, which are generated via a cellular engineering process, may lead to immunogenicity. Two available G-CSF drugs are produced by different expression systems: lenograstim, which is a glycosylated form, is expressed in mammalian cells, and filgrastim, which is a nonglycosylated form, is derived from prokaryotes (*E. coli*). Neither drug possesses the identical glycosylation pattern to that of the endogenous G-CSF protein in humans, but both possess the same biological activity as the endogenous human G-CSF protein^23^. Despite small differences were observed between recombinant and endogenous human G-CSF, the incidence of ADA production in patients treated with recombinant G-CSF has been reported in some clinical phase I studies^24^. This result suggested that recombinant proteins can induce immune responses; therefore, investigators focused on the development and safety assessment of biotherapeutics should make greater efforts to minimize patient risk, which is closely related to immunogenic potential.

Because development of almost all biotherapeutic drugs is goal for clinical application, it is derived from human-based proteins. It is a natural outcome which administration to animals, even though non-human primate, could evoke immune-related responses^25^. These immune responses are depending on several factors (e.g., species, dose route, dose level) and have relevance to hypersensitivity, vasculitis, and unwanted antibody production.

Nonclinical studies using animals may not consider to proper assessment for human risks^22^. Nevertheless, although issues related to insufficient translatability of the results from preclinical studies to clinical practice are present, still preclinical studies using animals are formally conducted prior to initiating clinical trials. The reason is that preclinical animal studies can provide previously unknown data regarding new drug-induced toxicities, and the results can be used to predict the efficacy, safety, and appropriate dose of a drug in humans^26^. Among preclinical findings, the occurrence of adverse effects caused by immunogenicity has become a major concern during the drug development process and evaluation of ADA is conducted to assist in the interpretation of the results and design of subsequent studies. Therefore, optimal analysis tools should be developed for the assessment of potential immunogenicity in the preclinical or earlier stage.

In conclusion, the results in this article suggested the need for pre-surveillance to evaluate the immunogenicity of GX-G3 and demonstrate the feasibility of these analytical procedures, which was importantly concerned with the implication of accurately interpretation of toxicokinetic data of recombinant G-CSF drugs in preclinical as well as clinical studies. This study might support the development of therapeutic biopharmaceutical, recombinant G-CSF, by providing useful information or translational alternatives to similar research fields.

## Materials and methods

### Materials

The drug GX-G3 was kindly supplied by Genexine, Inc. (Seongnam, Republic of Korea). Animal blank sera were prepared from 20 individual Sprague Dawley rats and 20 individual Cynomolgus monkeys. The anti-human G-CSF antibody and horseradish peroxidase (HRP)-conjugated streptavidin (streptavidin-HRP) were purchased from BD Pharmingen™ (San Diego, CA, USA). Carbonate-bicarbonate buffer, streptavidin-HRP, TMB substrate and Stop reagent were purchased from Sigma-Aldrich (St. Louis, MO, USA). Blocking buffer containing 1% BSA dissolved in 1 × PBS was obtained from Tech & Innovation (Seoul, Republic of Korea). 1 × PBS-T was purchased from Fluka (Buchs, Switzerland). Negative reference blank sera of healthy naïve rats and monkeys were obtained from BioChemed (Winchester, VA, USA).

### Preparation of assay reagents

Biotin-conjugated GX-G3 was prepared using SureLINK™ Chromophoric Biotin Labeling Kits from KPL Inc. (Gaithersburg, MA, USA). Both blocking buffer and biotin-conjugated GX-G3 were diluted in prepared 1 × PBS containing 1% BSA. 1 × PBS containing 0.05% Tween (PBS-T) was supplied as a washing buffer. All 20 blank sera of rats or monkeys were combined after NCO determination to generate a pooled blank serum sample as a blank matrix for each species. Three concentrations of positive control samples, high, middle, and low (0.4, 10, and 200 μg/mL, respectively, for rats; 0.4, 10, and 250 μg/mL, respectively, for monkeys), were prepared by spiking the positive control antibody into the pooled blank serum. A subset of positive control samples was stored frozen in a deep freezer to determine their long-term stability.

### Bridging ELISA procedures

MaxiSorp™ 96-well plates were coated with the drug GX-G3 in 50 mM carbonate-bicarbonate buffer (pH 9.6) overnight at 4 °C. The following day, the coated plates were washed three times with the washing buffer in a HydroFlex™ microplate washer (TECAN, Mannedorf, Switzerland) and blocked with blocking buffer at 37 °C for 2 h. After incubation, the blocking buffer was washed away three times. Negative control samples (NC), positive control samples (PC), and 1:20-diluted serum samples were added to the wells, and the plate was incubated for 90 min. Then, the wells were washed three times with washing buffer and treated with diluted biotin-conjugated GX-G3. Unbound material was washed away three times, and 100 μL of streptavidin-HRP diluted 1:50,000 in blocking buffer were dispensed into each well of the assay plate. This incubation proceeded for 50 min at 23 °C, and the wells were then washed five times. TMB substrate was added (100 μL/well), and the plate was incubated for 20 min in the dark. The HRP-substrate reaction was stopped by adding Stop reagent (100 μL/well), and the absorbance at 450 nm was measured using a SpectraMax® M3 Microplate Reader (Molecular Devices, Sunnyvale, CA, USA).

### Pre-study assay validation

#### Determination of negative cut-off (NCO) values

The NCO values were determined as follows. First, the absorbance at 450 nm was measured in all 20 blank animal serum samples (diluted 1:20) from rats and monkeys. The absorbance of each sample was evaluated independently three times, and the mean absorbance of each sample was used to determine the presence or absence of outliers using box plots in Analyse-it® software. Excluding outliers, an NCO value of blank sera was determined using the following formula when the Shapiro Wilk test confirmed normality of the data (p > 0.05): *NCO value* = *mean absorbance of blank sera* + (1.645 × S.D.)^21^. Then, the normalization factor was determined according to the following equation: *normalization factor* = *NCO value/mean absorbance*. Second, an equal volume of individual sera excluding outliers was mixed and used as a negative control sample for all experiments. The NCO value in each trial was determined by multiplying the specific normalization factor by the absorbance of the negative control sample.

#### Precision and sensitivity

High-, middle-, and low-concentration PC were analyzed in 5 independent assays. Mean absorbance values greater than the NCO value were classified as positive responses. Intra-assay precision was evaluated by calculating the % coefficient of variation (%CV, S.D./mean × 100) between the absorbance values from quintuplicate samples in each assay. Inter-assay precision was evaluated based on the % CV between the mean absorbance values of five independent assays. For the precision parameter, the default acceptance criteria for both intra- and inter-assay precision (%CV ≤ 25) were used.

Sensitivity was evaluated using samples spiked with a series of concentrations of the positive control antibody (0.24–729 ng/mL), and each sample was analyzed independently three times. In each assay, curve fitting was performed using a four-parameter regression model (positive control concentrations vs. absorbance). According to the curve fitting results, the sensitivity was determined as the concentration of the positive control antibody at which the absorbance was equal to the NCO value. All assays were accepted for analysis if the correlation coefficient (R^2^) was ≥ 0.98 based on the curve-fitting model.

#### Immuno-competition assay for confirmation of specificity

The high- and low-concentration PC were spiked with 200 μg/mL GX-G3 antigen or unrelated comparators (recombinant human growth hormone-hyFc or hyFc), and the mixtures were incubated for 1 h at room temperature (in a 23 °C incubator). Afterwards, the controls, such as PC not spiked with GX-G3 or unrelated comparators, were analyzed in comparison with the spiked samples. The difference in the absorbance values between groups was calculated using percentage of difference*.* For this parameter, the default acceptance criteria to identify a true positive (%difference ≥ 30 compared with the target drug GX-G3 and < 30 compared with unrelated comparatives) was adopted.

#### Estimation of drug interference

The low-concentration PC was spiked with GX-G3 antigen at a concentration of 250–8000 ng/mL. After incubation for 1 h at room temperature, the incubated sample was analyzed. The results were analyzed by curve fitting (GX-G3 concentration vs. absorbance) using a four-parameter regression model. In this analysis, drug interference was determined as the concentration of GX-G3 at which the absorbance value was equal to the NCO value; this concentration represented an approximate limit of drug tolerance for positivity discrimination. All assays were accepted for analysis if the correlation coefficient (R^2^) was ≥ 0.98 based on the curve-fitting model.

#### Stability

Stability of the high- and low-concentration PC was evaluated under two conditions: 5-repeated cycles of freeze (at -80 ℃ for minimum 12 h) and thaw (at room temperature for minimum 2 h), and long-term freeze at − 80 ℃ for 12 weeks. These PCs were compared with the control samples prepared on the day of analysis. The percentage of difference between stability samples and control samples was calculated using the ratio values of PCs which were adopted within %difference ≤ 25 when compared with the result in precision.

### Toxicological evaluation and sample collection

The GLP-compliant 4-week repeated-dose toxicity studies with a 4-week recovery period were performed using rats and monkeys. Crl:CD (SD) rats and cynomolgus monkeys were purchased from Orient Bio Inc. (Gyeonggi, Republic of Korea) and Nafovanny (Tam Phuoc Hamlet, Log Thanh District, Dong Nai Province, Vietnam), respectively. The animals were housed in a temperature- (23 ± 3 °C) and relative humidity- (50 ± 10%) controlled room. Lighting was adjusted automatically providing a 12/12 h light/dark cycle. All animal experimentation was carried out in accordance with the principles of the American Association for Accreditation of Laboratory Animal Care (AAALAC, accredited since 1998) in fully accredited facilities. The procedure of animal husbandry and all experimental protocols in rats and monkeys were approved by the Institutional Animal Care and Use Committee (IACUC) in the Korea Institute of Toxicology (Approval No. 1301-0019 for rat study and 1208-0254 for monkey study). These studies were carried out in compliance with the ARRIVE guidelines. As shown in Table 3, animals were assigned to 4 groups, which were subcutaneously administered GX-G3 at doses of 0 (vehicle control), 1 (T1), 3 (T2), or 10 (T3) mg/kg once a week for 4 weeks. Rats and monkeys were assigned into four main groups of 3 animals per sex and two recovery groups (vehicle control and T3 groups) of 2 animals per sex. For the analysis of anti-GX-G3 antibodies, blood was collected from rats on weeks 0 (pre-dose), 2 (Day 14), 4 (Day 28), and 8 (Recovery day 29) and from monkeys on weeks 0 (Day 1), 1 (Day 8), 3 (Day 22), 4 (Day 29) and 8 (Recovery day 30). These ADA samples were taken before administration on dosing day for trough levels, For the analysis of serum concentration of GX-G3, blood was collected from tail vein of rats and femoral vein of monkeys on 0, 6, 24, 36, 72, 120 and 168 h after administration on Day 1 and Day 22. The blood samples were collected into serum separation tubes, incubated at room temperature for approximately 30 min, and centrifuged at 13,200 rpm for 5 min. The separated serum was stored in a deep freezer (at − 80 °C) for analysis.

**Table 3:** Experimental design of the current toxicity study of GX-G3 treatment in rats and monkeys.

Species	Group	Dose (mg/kg)	Number of animals (male/female)	
			Week 0, 2, and 4	Week 8
			(Dosing day 1^a^, 14, and day 28)	(Recovery day 29)
Rats	Vehicle control	0	3/3	3/3
	T1	1	3/3	3/3
	T2	3	3/3	3/3
	T3	10	3/3	3/3

Species	Group	Dose (mg/kg)	Week 0, 1, 3, and 4	Week 8
			(Dosing day 1, 8, 22, and 29)	(Recovery day 30)
Monkeys	Vehicle control	0	3/3	2/2
	T1	1	3/3	–
	T2	3	3/3	–
	T3	10	3/3	2/2

^a^Rat samples for 0 h on Day 1 (Week 0) collected from pre-dosing status.

### Sample analysis for anti-GX-G3 antibody

#### Screening assay

Screening assays were first performed on the samples to determine whether each sample was positive for anti-GX-G3 antibodies. The normalization factor was multiplied by the absorbance of the negative control sample to calculate the NCO value: *NCO value* = *mean absorbance of negative control sample* × *normalization factor.*

#### Confirmation assay

Confirmation assays were performed using the immune-competition method to discriminate true positive samples among positively screened samples in the previous step. According to the pre-study assay validation results, the positively screened samples were pre-treated with GX-G3 for 1 h at room temperature and then analyzed. True and false positive samples were discerned based on a %difference ≥ 30.

#### Titer determination

Samples that were determined as true positives were prepared as serial three-fold dilutions (from 20- to 393660-fold), and the antibody titer was analyzed. The greatest dilution that displayed a positive response (≥ NCO value) was identified for each sample.

#### Statistical analysis

Before evaluating the statistical differences in antibody titers, all data were tested for a normal distribution. Because the antibody titer was not normally distributed based on Levene’s test, non-parametric Kruskal–Wallis and Mann–Whitney tests were used to assess the statistical differences between groups. All calculations were performed using SPSS 17.0 software, and p < 0.05 was considered to indicate a significant difference.

### Toxicokinetic analysis

Male and female rats and monkeys were assigned to three treatment groups (n = 6 per group; three males and three female animals). On the day 1 and day 22 of dosing, time-coursed samples were measured using a validated detection method with sandwich ELISA (data not shown due to the limited contents in the manuscript). After the measurement, total GX-G3 serum concentration–time profiles were used to estimate the following TK parameters in rat and cynomolgus monkey, using non-compartmental analysis in WinNonlin® (software version 5.2, Certara, USA). The maximum serum concentration (C_max_) was obtained from individual serum concentration vs. time profiles. The area under the serum concentration vs. time curve from 0 h to infinity was obtained by linear trapezoidal method. Terminal half-life (T_1/2_) was calculated using terminal elimination rate constant 0.693. The total clearance (CL) was calculated as Dose/AUC_inf_. For toxicokinetic analysis of rat and monkey data, each animal was analyzed separately and results for each dose group were summarized as mean value and no formal statistical analysis was performed to determine the significance of the difference among the exposure groups.
